# Toward understanding scarless skin wound healing and pathological scarring

**DOI:** 10.12688/f1000research.18293.1

**Published:** 2019-06-05

**Authors:** Sanna-Maria Karppinen, Ritva Heljasvaara, Donald Gullberg, Kaisa Tasanen, Taina Pihlajaniemi

**Affiliations:** 1 Oulu Center for Cell-Matrix Research, Faculty of Biochemistry and Molecular Medicine, University of Oulu, Oulu, Finland; 2 Biocenter Oulu, University of Oulu, Oulu, Finland; 3 Department of Biomedicine, University of Bergen, Bergen, Norway; 4 Centre for Cancer Biomarkers (CCBIO), University of Bergen, Bergen, Norway; 5 Oulu Center for Cell-Matrix Research, PEDEGO Research Unit, University of Oulu, Oulu, Finland; 6 Medical Research Center and Department of Dermatology, University of Oulu/Oulu University Hospital, Oulu, Finland

**Keywords:** wound healing, keloid, hypertrophic scar, myofibroblast, extracellular matrix, fibrosis

## Abstract

The efficient healing of skin wounds is crucial for securing the vital barrier function of the skin, but pathological wound healing and scar formation are major medical problems causing both physiological and psychological challenges for patients. A number of tightly coordinated regenerative responses, including haemostasis, the migration of various cell types into the wound, inflammation, angiogenesis, and the formation of the extracellular matrix, are involved in the healing process. In this article, we summarise the central mechanisms and processes in excessive scarring and acute wound healing, which can lead to the formation of keloids or hypertrophic scars, the two types of fibrotic scars caused by burns or other traumas resulting in significant functional or aesthetic disadvantages. In addition, we discuss recent developments related to the functions of activated fibroblasts, the extracellular matrix and mechanical forces in the wound environment as well as the mechanisms of scarless wound healing. Understanding the different mechanisms of wound healing is pivotal for developing new therapies to prevent the fibrotic scarring of large skin wounds.

## Introduction

Intact healthy skin protects the body from outside threats; therefore, proper wound healing is an essential process in response to tissue damage ^1– 3^. The formation of a scar comprises a crucial part of normal mammalian tissue repair but defects in its resolution can lead to excessive accumulation of extracellular matrix (ECM) in the tissue and cause pathological scarring. In addition, overproduction of ECM components and tissue hardening characterise other fibrotic conditions of the skin, namely scleroderma affecting limited areas of the skin and systemic sclerosis affecting the whole skin and internal organs ^4^. Fibrosis also affects many other organs, such as the heart, liver, kidney, and lungs, leading to severe dysfunction of these tissues. Aberrant skin wound repair leading to chronic non-healing wounds and pathological scarring and fibrosis after severe trauma affect millions of people worldwide, but effective cure or therapeutics for adverse scarring are still lacking. However, because cutaneous wound healing and fibrosis are extensively studied, the obtained data can be exploited for developing therapies for the related pathological conditions in many other tissues as well.

**Figure 1.**
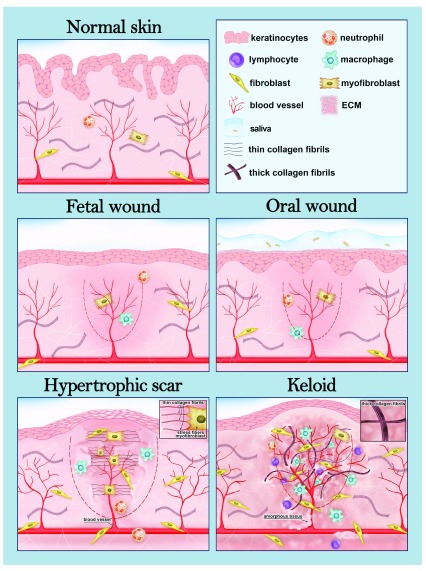
Foetal and oral wound healing and different types of fibrotic skin scars. A schematic drawing showing the structures of normal skin, scarless foetal and oral wounds, and the two main types of fibrotic cutaneous scars. The wound area is depicted with a dashed line. Foetal and oral wound healing share many characteristics. For example, these wounds contain a low number of myofibroblasts and extracellular matrix (ECM) does not accumulate in the wound bed. In addition, the inflammatory reaction is weak, which is manifested here by the low number of inflammatory cells in the wound. In oral wounds, saliva offers a humid environment with microbes, which is suggested to promote oral wound healing. The hypertrophic scar is limited to the area of the original wound and contains plenty of contracting myofibroblasts, which adhere to ECM via focal adhesion-like structures. Thin collagen fibres in the ECM are orientated in parallel to the cutaneous epithelia (insert). The keloid scar transcends the edges of the wound and extends into the surrounding skin. The inflammatory reaction is strong, dermal fibroblasts proliferate actively and thick hyalinised collagen bundles are orientated randomly (insert). Chronic inflammation persists, and angiogenesis is active in the keloids.

Tissue damage repair aims at restoring tissue integrity and consists of complex and tightly regulated biological processes involving extensive cooperation of several cell types, growth factors and the cytokines secreted by them and the surrounding ECM ^5^. Adult skin wounds heal by scarring, which restores the barrier function of the skin and thereby prevents the body from dehydration and protects wounds from infections ^5^. A normal scar is composed of loose fibrous connective tissue and is slowly remodelled during the healing process to become stronger; however, it remains weaker and functionally deficient compared with uninjured tissue ^5^. Chronic inflammation of the dermis and uncontrolled function of activated connective tissue cells, myofibroblasts, may lead to abnormal overgrowth of the scar, resulting in a hypertrophic scar or a keloid with an excess of ECM proteins ^6^ ( Figure 1). These two pathological types of scars have a different aetiology and unique and distinct structural and molecular characteristics, as will be discussed below in more detail. Promoting wound healing without excessive scarring is important in terms of both function and aesthetics.

Notably, foetal skin wounds heal without a visible scar until gestational week 24. Even some adult tissues, including the oral mucosa, heal with minimal scar formation ^7^. We propose that a detailed understanding of the mechanisms of foetal and oral wound healing will increase our understanding of the scarless repair process in general and help in developing therapies for wounds and fibrotic scars ^8– 11^.

In this review, we briefly summarise the processes and key mechanisms of normal and scarless wound healing and the features and pathogenesis of hypertrophic and keloid scars. We also discuss the impact of myofibroblasts and mechanical stress as well as cell–cell interactions in cutaneous wound healing and fibrosis, uncovering recent advances in the field.

## Normal wound healing

Cutaneous wound healing in adults is well understood and consists of four partly overlapping phases: haemostasis, inflammation, proliferation, and remodelling ^5, 12, 13^. The three latter phases determine whether the wound heals normally or whether an aberrant healing process leads to the excessive production of ECM proteins and to fibrosis ^6, 11, 12, 14^.

Haemostasis starts with the formation of a blood clot that arrests bleeding and protects the wound area from microbial invasion ^5, 14^. The formed fibrin network stores growth factors and serves as a platform for migrating vascular cells, leukocytes and fibroblasts. An inflammatory reaction is induced by growth factors (principally by the transforming growth factor beta 1, or TGFβ1), cytokines (for example, interleukins IL-1 and IL-6) and chemokines (for example, chemokine C-X3-C ligand 1, or CX3CL1) released from platelets and damaged keratinocytes ^5, 12, 14^. Infiltrating inflammatory cells eliminate microbes and produce oxygen radicals and proteinases to fight pathogens. They also secrete growth factors, cytokines and chemokines that activate the proliferation phase, which comprises neovascularisation, formation of granulation tissue, and re-epithelisation ^5, 14^. The formation of new blood vessels is activated by cytokines and vascular growth factors—for example, vascular endothelial growth factors (VEGFs) and basic fibroblast growth factor (bFGF)—and is important for the progression of healing since the newly formed wound area suffers from hypoxia and lack of nutrients. Dermal fibroblasts start to proliferate and produce large amounts of ECM components, forming a temporal connective tissue called granulation tissue rich in capillaries, macrophages and fibroblasts. Re-epithelisation starts when basal keratinocytes and regenerative epidermal stem cells derived from interfollicular epidermis, hair follicles and sebaceous glands ^15– 17^ divide and differentiate in the wound edges and migrate along the surface of the granulation tissue to cover it and form the outermost protective layer of the wound. Keratinocytes lying in the wound edge produce matrix metalloproteases (MMPs) such as MMP-1, which degrades ECM and decreases adhesion of keratinocytes, thereby promoting their migration. Mechanical stress, resulting from ECM deposition, and secreted factors, primarily TGFβ1, activate many different cell types, such as fibroblasts, pericytes, adipocytes, resident mesenchymal progenitor cells, and bone marrow–derived mesenchymal stem cells, to form a heterogenic population of myofibroblasts that actively proliferate and secrete ECM proteins such as collagens I and III and fibronectin ^6, 18, 19^. Myofibroblasts attach to the ECM via integrins and contract through the alpha-smooth muscle actin (αSMA)-rich stress fibres, effectively narrowing the wound area. The last phase of wound healing, maturation, is characterised by the apoptosis of myofibroblasts and other cells and by the remodelling of the connective tissue (for example, through action by MMPs), all in all resulting in a tight scar structure ^12, 13^. Finally, the ratio of fibrillar collagen I to collagen III returns to the level of normal skin (5:1) from that in the temporal wound matrix (2:1), and fibril size increases, corresponding to the characteristics of a healthy dermis ^5, 14^.

## Pathological scarring: hypertrophic scars and keloids

Whereas superficial epidermal skin damage heals efficiently, the healing of deeper dermal wounds may lead to abnormal scar overgrowth and formation of two different types of fibrotic skin disorders: hypertrophic scars or keloids. The mechanisms of scar formation, the characteristic features and partially the treatments of these two types of pathological scars are different ^20– 24^. In clinical settings, despite intensive research aiming to unravel the molecular and morphological differences of hypertrophic scars and keloids, their separation remains challenging and sometimes an injured area contains features from both scar types ^24^.

Hypertrophic scars are defined as raised, erythematous, pruritic lesions that do not extend beyond the boundaries of the original wound area ( Figure 1). They usually appear near the joints or other areas exposed to stretching ^9, 10^, grow rapidly during 4 to 12 weeks after wounding and tend to mature and flatten over time. Keloids protrude from the original wound site, invading the surrounding skin ( Figure 1), and they may appear even years after the trauma, develop slower than hypertrophic scars and almost never regress but continue to grow like a benign fibroproliferative tumour ^9, 10, 25^. They arise from different kinds of skin damage such as scratches, insect bites, vaccination, perforation, acne, surgical wounds, and burn injuries, and they usually appear in skin areas that lack hair follicles, namely in the neck, chest, shoulders, upper back, auricles and abdomen ^10, 25^. Keloids are most common among individuals of African or Asian ancestry and tend to have a strong but poorly characterised genetic background ^8^. Hypertrophic and keloid scars also differ structurally, the main disparities existing in the size and architecture of the collagen fibres ( Figure 1) and their cellular composition. However, the differences in the orientation of collagen fibres in various scar types have also been questioned ^26^.

The pathogenesis of both hypertrophic scars and keloids is poorly understood ^9, 11^. According to the current understanding, scar overgrowth is thought to be controlled by the inflammation in the reticular dermis, with accumulation of inflammatory cells and fibroblasts to the scar area. In addition, neovascularisation and the formation of collagen fibres are active ^25, 27^. In the keloids, the inflammatory reaction is strong, and the levels of pro-inflammatory cytokines, such as IL-1, IL-6, and tumour necrosis factor alpha (TNFα), are high. This promotes chronic inflammation and results in the protrusion of the keloid beyond the original wound area. Mast cells and T and B lymphocytes are prominent in keloid scars. In addition, alternatively activated type M2 macrophages, which have been associated with fibroblast activation, collagen formation and fibrogenic disorders ^28^, accumulate in keloids. However, the exact roles of macrophages in the formation of pathological scars are incompletely understood and the contribution of these cells will be discussed later in this review. In hypertrophic scars, the inflammatory reaction is weaker than in keloids because of differences in the intensity, frequency and duration of the inflammation in the reticular dermis. For example, immune cells are less frequent than in keloids ( Figure 1), and expression of several inflammatory genes (for example, *TNFα*, *IL-1*, and *IL-10*) is decreased in human hypertrophic scars when compared with normally healing scars ^29^.

Other key factors affecting the pathogenesis of hypertrophic scars and keloids are fibroblasts, growth factors, cytokines and ECM remodelling ^6, 30– 33^. In keloids, fibroblasts are more sensitive to TGFβ1 than in normal skin, and their density and proliferation rate are high, whereas apoptosis is lower. The constant presence of αSMA-positive contracting myofibroblasts is typical for hypertrophic scars. Fibroblasts and myofibroblasts, stimulated by several growth factors such as TGFβ, platelet-derived growth factor (PDGF), and insulin-like growth factor (IGF), produce high amounts of collagen and other ECM components, thereby accelerating the formation of abundant fibrotic tissue in pathological scars ^31, 32^. Collagen synthesis is estimated to be 7-fold higher in hypertrophic scars and up to 20-fold higher in keloids compared with normal skin ^30^. In hypertrophic scars, the ratio (6:1) between collagen I and collagen III is lower than it is in keloids (17:1) and thus is closer to the value of normal skin (5:1). In keloids, the amount of collagen cross-links is twice that of hypertrophic scars, resulting in the formation of thick collagen bundles. In addition, the levels of many other ECM components, such as hyaluronic acid, fibronectin, tenascin and MMP-19, are increased in both pathological scar types ^31– 33^, but their relative expression and the localisation in the scar area may vary in between ^24^.

## Myofibroblasts and mechanical forces in cutaneous wound healing and fibrosis

Deposition of ECM and temporary scar formation are part of a normal healing process; however, accumulation of abnormally organised stiff ECM that replaces the normal tissue is a characteristic feature of fibrotic scars. The most important cells promoting scarring and fibrosis are activated fibroblasts—myofibroblasts—that secrete high amounts of ECM components with abnormal structural and mechanical properties and with an altered capacity to bind growth factors ^34– 36^. Ultimately, excessive ECM accumulation and cross-linking lead to increased tissue stiffness and pathological scarring, causing impaired function of the skin ^34^.

The mechanical microenvironment affects scar contracture, and if the contraction continues after healing, it results in a poor functional and cosmetic outcome ^2, 3, 34, 36^. Fibroblasts are sensitive to exogenous mechanical forces, which trigger the upregulation of several fibrotic genes, encoding proteins such as TGFβ, αSMA and collagen I, through different mechanoreceptors such as integrins, growth factor receptors, G protein–coupled receptors and ion channels ^2, 37^. The cellular contractile forces in the activated myofibroblasts are critical to maintain scar contracture through their adhesion to the ECM. The integrin-focal adhesion kinase (FAK) pathway is central in regulating skin mechanotransduction. FAK is activated in response to mechanical forces during wound healing, and it affects intracellular signalling by numerous downstream factors, such as PI3K and MAPK kinases, which mediate fibrotic responses ^2, 36, 38^. Decreased FAK signalling has been observed in non-healing wounds, whereas excessive FAK activation leads to the formation of hypertrophic scars. In addition, FAK degradation in diabetic ulcers has been associated with delayed wound healing and abnormal scar architecture ^39^. Increased tension in the wound area induces hypertrophic scarring ^36, 37^, and scarless foetal wounds are known to have a lower resting stress compared with post-partum wounds ^2^.

Therapeutic possibilities for preventing pathological skin scarring are still limited and have been focused mainly on reducing inflammation and contraction of the wound ^2, 21– 23^. TGFβ1, as an inducer of myofibroblast differentiation, is considered a potential therapeutic target for the prevention of pathological scars ^9, 21, 40^. In addition, several other secreted factors, such as connective tissue growth factor (CTGF), PDGF, IGF, VEGF, and IL-6, are known to promote myofibroblast differentiation ^37^, whereas others, such as FGF, epidermal growth factor, interferon gamma and IL-10, have the opposite effect. Other recently described factors that affect myofibroblasts and fibrosis include integrin α11β1, a mediator of pro-fibrotic signals in dermal fibroblasts; cartilage oligometric matrix protein (also known as thrombospondin-5), which has a role in exporting collagen from fibroblasts; and integrin-linked kinase, a mechanotransducer and signal transmitter that controls TGFβ1 secretion ^34^. The absence or inhibition of these proteins in mice has been shown to reduce fibrosis and thus has been suggested to be a suitable target for anti-fibrotic therapies ^34^. Since scar formation is reduced upon decreasing mechanical forces in the wound area, scar-reducing therapies use mechanical off-loading ^2, 41^. Several biochemical signals related to mechanical tension have been reported to alleviate skin scarring, such as TGFβ1 inhibition, addition of TGFβ3 or down-regulation of connexin 43 ^42^. Currently, three different off-loading techniques are used in humans: silicone gel sheets, paper tape and embrace advanced scar therapy ^2^. However, specific molecular mechanisms behind the therapies that use reducing tension in the wound area are still incompletely understood ^2^ and more knowledge is needed to improve the current therapies.

## The emerging roles of cell–cell interactions during wound healing and fibrosis

Wound healing involves several cell types, and unravelling their roles and mutual interactions is important for understanding the different phases of wound closure. For example, hair follicle stem cells interact with fibroblasts through the Wnt/β-catenin pathway to convert them to myofibroblasts to help in wound contraction ^36^. In addition, the importance and therapeutic potential of macrophages in the wound healing process have been highlighted in recent years ^3, 36, 43^. A recent study reports an interesting cross-talk between myofibroblasts and macrophages during skin repair, indicating the potential role of adipogenic cells in wound healing and scarring ^44^. Lineage tracing and flow cytometry revealed different subpopulations of myofibroblasts in adult mouse wounds, including those derived from CD26-expressing adipocyte precursors (APs) and others from cells with high CD29 (β1 integrin) expression ^44^. Growth factors, such as PDGF-C and IGF1, secreted by CD301b ^+^ M2-type macrophages were shown to selectively stimulate the proliferation of the AP-derived myofibroblasts but no other myofibroblast subsets, contributing to myofibroblast heterogeneity. In the wounds of aged mice or in experimentally induced mouse skin fibrosis, the AP-derived myofibroblasts and CD301b ^+^ macrophages were significantly reduced, and the CD29 ^+^ pool was increased when compared with a normal wound healing environment. Interestingly, in keloids, the numbers of CD301b ^+^ macrophages and CD26 ^+^ AP-derived myofibroblasts are also increased ^45^. Another recent study showed that, during lung fibrosis, cadherin-11 mediates the adhesion between macrophages and myofibroblasts, promoting the activation of myofibroblasts and supporting their activity by targeting the macrophage-produced TGFβ to myofibroblasts ^46^. Taken together, these results suggest that the presence of distinct myofibroblast populations in different fibrotic microenvironments provides possibilities for targeting specific subpopulations of cells in anti-fibrotic therapies aiming at scarless wound healing.

## Scarless wound healing: foetal and oral wounds

Foetal and oral mucosal wound healing have been regarded as key models for scarless healing. However, despite intensive studies over the years, the mechanisms of both healing processes remain largely unknown ^47, 48^. Several studies have described mechanistic differences between scar-forming and scarless wound healing; however, a better understanding of the key cellular and molecular factors regulating these pathways would benefit the development of therapeutic tools for pathological cutaneous scars ^47, 48^.

### Foetal wound healing

Skin wounds arising during the first and second trimesters of pregnancy heal perfectly without forming any scar ^5, 10, 47^. Foetal wound healing does not follow the four-step process of adults. In addition, the wounds do not contract, ECM components do not accumulate, and granulation tissue is not formed ( Figure 1). Also, skin appendages such as hair follicles and sweat glands re-form perfectly during foetal wound healing in contrast to the adult scar that forms without any skin appendages ^5, 47, 48^. Recent studies have revealed that adult skin lacks, but foetal/neonatal skin includes, a subpopulation of fibroblasts in the upper part of the dermis which is needed for hair follicle regeneration after wounding ^18, 49^. Thus, foetal wound healing is considered to be more like a regenerative process, in which the damaged components are replaced and the tissue returns to its normal state, than a reconstructive healing (termed repair) aiming to restore the tissue architecture and structure with fibrotic healing and scar formation. The signalling pathways regulating the development and growth of the skin are thought to play a key role in this regenerative healing ^50^.

Multiple cell- and molecular-level distinctions exist between the foetal and adult wound healing processes in mammals ^1, 12, 20, 47, 48, 50^. One of these is a weak inflammatory reaction in foetal wounds which is due to an immature immune system. The degree and duration of the inflammatory reaction as well as the composition of the immune cells influence the final healing outcome ^51^. Neutrophils, macrophages and mast cells are involved in skin scarring ^52^, and foetal wounds have a lower amount of these cells; furthermore, the cells are less differentiated. Their growth factor and cytokine profiles are different as well. For example, the pro-inflammatory cytokines IL-6 and IL-8 are produced for several days in human adult wounds but only for a relatively short period during the foetal stage ^20^. Moreover, the levels of anti-inflammatory cytokines, especially IL-10, which inhibits neutrophil and macrophage infiltration and prevents scarring, are high. The crucial role of the inflammatory cells in scarring is further demonstrated in mutant mice that lack the essential hematopoietic transcription factor PU.1 and thus also neutrophils, macrophages, and mast cells: the adult skin wounds in these mice heal efficiently without scarring ^53^. These observations suggest that the inflammatory reaction is a crucial mediator promoting fibrosis and scarring in adults but its role is still unclear. However, since fibrosis has been observed in the wounds of several immunodeficient mouse lines, it is known that scarring does not depend only on the lymphocyte-mediated response. Furthermore, some studies suggest a supportive role for inflammatory cells during repair and regeneration. For example, knockdown of macrophages, but not neutrophils, resulted in impaired healing in rabbits, and in lower vertebrates the depletion of macrophages was reported to result in failing regeneration (for example, regarding the limb in salamander or the tail fin in zebrafish) ^51^.

Another important difference between adult and foetal wounds is the production of ECM components, ECM-degrading enzymes and tissue inhibitors of metalloproteinases (TIMPs) ^30, 47^. There are disparities in the size and orientation of the collagen fibres and in the mechanical properties of the ECM, even between healthy adult and foetal skin. In foetal skin wounds, the number of αSMA-positive contracting myofibroblasts is very low ( Figure 1) and fibroblasts migrate faster to injured sites, simultaneously proliferating and producing ECM that more closely resembles the ECM of developing skin and maintains this normal architecture and strength during the healing process ^47^. Therefore, foetal scarless wounds also contain more collagen III than collagen I (collagen III comprises 30 to 60% of total collagen in foetal skin and only 10 to 20% in adult skin) and more hyaluronic acid ^2, 47, 54^. The ratio of MMPs to TIMPs is high in foetal wounds; thus, the ECM components are actively degraded, favouring remodelling over accumulation of the ECM in the wound area ^30, 47, 48, 55^. In addition, the amounts of some adhesion proteins, such as tenascin and fibronectin, which are more prominent in healing wounds than in healthy skin, are higher in foetal wounds, supporting cell attraction and migration to the sites of injury thereby facilitating scarless wound repair ^36, 47^. Finally, the expression of the collagen cross-linking enzyme lysyl oxidase (LOX), associated with pathogenesis of fibrotic diseases, is lower ^47^.

Of particular interest is the difference in the expression of the TGFβ family of growth factors that are involved in every step of wound healing dealing with processes such as cell proliferation, differentiation, angiogenesis, ECM production and modulating immune response ^40, 56^. Different isoforms (TGFβ1, 2 and 3) bind to the same receptors (TGFβRI and II) and signal through both canonical and non-canonical pathways. There is emerging evidence that the biological roles of these isoforms are different, but their mechanisms of action have remained partly unclear, although some evidence for differences in signal transduction, including transcriptional regulation of target genes, has been suggested ^40, 56^. In addition, the expression of TGFβ isoforms varies during different phases of wound healing. Foetal skin wounds, completely opposite to adult wounds, show high levels of TGFβ3 and low levels of TGFβ1 and TGFβ2 ^1, 47, 56^. In foetal wounds, TGFβ3 signalling decreases the number of macrophages and monocytes and the expression of collagens and fibronectin in the wound bed ^47^. In clinical experiments, TGFβ3 injected into adult wounds was shown to reduce scar formation and induce collagen organisation equivalent to that of normal skin ^9, 57^. Moreover, blocking the function of TGFβ1 and TGFβ2 (for example, with neutralising antibodies) reduced the severity of scarring, suggesting these growth factors as good candidates in scar prevention. Nevertheless, attempts to prevent scar formation by targeting different TGFβ isoforms have resulted in contradictory results so far, and many successful preclinical models have failed in clinical trials ^9, 21, 57^.

Indeed, there are also some contradictory preclinical data such as the use of TGFβ3 in a rabbit ear wound model which showed faster healing but no decrease in the scarring ^40^. In clinical trials, a TGFβ1 inhibitor and a monoclonal antibody against TGFβ1 were found to be ineffective in the treatment of scleroderma and systemic sclerosis, and human recombinant TGFβ3 failed in a phase III clinical trial on human scarring ^57^. In addition, a recent comparison of human hypertrophic scars with normally healing scars showed increased and persistent expression of the *TGFβ3* gene; surprisingly, there was no difference in *TGFβ1* expression ^29^. This result conflicts with the view of differing TGFβ signalling between adult and foetal wounds and could partly explain the failure of TGFβ3 to prevent fibrosis in clinical trials ^1, 9, 21, 47, 57^. Moreover, in hypertrophic scars, the expression of TGFβ1 and TGFβ2 is lower compared with keloids whereas the expression of TGFβ3 is higher. These observations, combined with several other examples, point out the importance and complexity of different TGFβ isoforms and their relative ratios in regulating wound healing processes and different forms of scarring. The context-dependent outcome of TGFβ signalling and its pleiotropic effects and the large number of different factors involved, such as the balance between ligands, different cells, signalling mediators, activated downstream pathways and ECM stiffness, make therapeutic targeting extremely challenging. Therefore, it is probable that effective therapies in the future will be based on a combination of different factors rather than any single molecular target ^40, 56, 58^.

### Oral wound healing

Oral mucosal wounds heal rapidly with minimal scar formation, sharing some characteristics of the foetal healing process ( Figure 1). Compared with cutaneous wounds, oral wounds differ in the production of ECM components; for example, hyaluronan, tenascin and fibronectin are highly expressed in both oral and foetal wounds, and the ratios of collagen III to collagen I and MMPs to TIMPs are high ^1, 7, 59^. Furthermore, the number of growth factors and cytokines as well as bone marrow–derived cells and blood vessels and levels of mediators contributing to immune and profibrotic responses show differences. For example, the numbers of neutrophils, macrophages and T cells are reduced in oral wounds ( Figure 1). Several inflammatory cytokines, such as IL-23, IL-24 and inflammatory response–inducing interferons, are absent and the pro-inflammatory cytokines IL-6 and IL-8 are only briefly expressed ^7^. In oral wounds, compared with cutaneous wounds, the levels of VEGFs are reduced and thus angiogenesis is less active. Myofibroblast differentiation may also differ between oral and dermal wounds. Although oral wounds have more αSMA-positive myofibroblasts with effective contraction capacity and a high proliferation rate, the cells are less responsive to TGFβ1, which is also less expressed in oral wounds, than adult dermal fibroblasts ^7, 59– 62^.

Re-epithelialisation is faster in oral than in cutaneous wounds, suggesting greater proliferative capacity for oral keratinocytes ^57^. Human oral and cutaneous keratinocytes show differences in their morphology and differentiation and in their gene expression profiles. Moreover, a recent study showed that biopsies from human oral and cutaneous repair sites exhibit specific transcriptional signatures during wound healing, highlighting the reduced differentiation capacity and inflammatory response of oral mucosa compared with skin ^63, 64^. In addition, it is suggested that environmental factors, primarily saliva with high levels of microbes, support oral wound repair ^7^. It should be noted that addressing the mechanisms behind oral wound healing has resulted in contradictory findings in certain oral regions, and the healing outcomes vary considerably, ranging from scarless healing to extensive scar formation ^65^. For example, palatal and gingival wounds have been reported to heal without scarring in one study but another study reported rigid scar formation in palatal wounds ^7^.

## Conclusions

Scar tissue is formed mainly of fibrillar collagen produced by myofibroblasts. Factors that are important in the activation of myofibroblasts include the availability of active growth factors, such as TGFβ1 and PDGF; the activation of inflammatory signalling; and a mechanical stress response. Currently, the key clinical treatments used for preventing pathological scarring are silicone gels or surgical operations to reduce wound contraction as well as medication that decreases inflammation in the wound area. Despite the already well-understood basic molecular mechanisms of wound healing, knowledge about hypertrophic and keloid scars is somewhat contradictory, which complicates the development of efficient wound care for different types of fibrotic scars. Part of the problem lies in the imperfect *in vitro* wound healing assays that lack the standardisation of experimental variables such as the used cell culture surfaces, which can range from plastic to biological scaffolds with variations in ECM composition and stiffness, as well as limited correlation between rodent, rabbit and pig wound healing models and human wound physiology ^29^. In addition, understanding the details of the coordinated actions and plasticity of different cell types in the wounds, regulation of growth factor and cytokine synthesis, and changes in ECM dynamics during various phases of wound healing and scar formation is still insufficient. However, the various mechanisms are extensively studied in the context of both scarless and fibrotic wound healing, and clinical trials—for example, with human recombinant TGFβ3, PDGF-B, fibrin platelet-rich plasma concentrate, estradiol and erythropoietin among many others—are ongoing ( https://clinicaltrials.gov/ and https://www.clinicaltrialsregister.eu/). Hopefully, these clinical trials and basic science research efforts will lead to a better understanding and treatment of both cutaneous pathological scars and a wide spectrum of fibrotic diseases in other tissues.
